# From pixels to prognosis: Leveraging radiomics and machine learning to predict IDH1 genotype in gliomas

**DOI:** 10.1007/s10143-025-03515-z

**Published:** 2025-04-29

**Authors:** Asli Beril Karakas, Figen Govsa, Mehmet Asim Ozer, Huseyin Biceroglu, Cenk Eraslan, Deniz Tanir

**Affiliations:** 1 Department of Anatomy, Faculty of Medicine, Kastamonu University, Kastamonu, 37200 Turkey; 2 Department of Anatomy, Faculty of Medicine, Ege University, Izmir, Turkey; 3 Department of Neurosurgery, Faculty of Medicine, Ege University, Izmir, Turkey; 4 Department of Radiology, Faculty of Medicine, Ege University, Izmir, Turkey; 5 Department of Management Information Systems, Faculty of Economics and Administrative Sciences, Kafkas University, Kars, Turkey

**Keywords:** Glioma, IDH1, Radiomics, Machine learning (ML), K-Nearest Neighbor (KNN), Support Vector Machine (SVM), Magnetic Resonance Imaging (MRI)

## Abstract

Gliomas are the most common primary tumors of the central nervous system, and advances in genetics and molecular medicine have significantly transformed their classification and treatment. This study aims to predict the IDH1 genotype in gliomas using radiomics and machine learning (ML) methods. Retrospective data from 108 glioma patients were analyzed, including MRI data supported by demographic details such as age, sex, and comorbidities. Tumor segmentation was manually performed using 3D Slicer software, and 112 radiomic features were extracted with the PyRadiomics library. Feature selection using the mRMR algorithm identified 17 significant radiomic features. Various ML algorithms, including KNN, Ensemble, DT, LR, Discriminant and SVM, were applied to predict the IDH1 genotype. The KNN and Ensemble models achieved the highest sensitivity (92-100%) and specificity (100%), emerging as the most successful models. Comparative analyses demonstrated that KNN achieved an accuracy of 92.59%, sensitivity of 92.38%, specificity of 100%, precision of 100%, and an F1-score of 95.02%. Similarly, the Ensemble model achieved an accuracy of 90.74%, sensitivity of 90.65%, specificity of 100%, precision of 100%, and an F1-score of 95.13%. To evaluate their effectiveness, KNN and Ensemble models were compared with commonly used machine learning approaches in glioma classification. LR, a conventional statistical approach, exhibited lower predictive performance with an accuracy of 79.63%, while SVM, a frequently utilized ML model for radiomics-based tumor classification, achieved an accuracy of 85.19%. Our findings are consistent with previous research indicating that radiomics-based ML models achieve high accuracy in IDH1 mutation prediction, with reported performances typically exceeding 80%. These findings suggest that KNN and Ensemble models are more effective in capturing the non-linear radiomic patterns associated with IDH1 status, compared to traditional ML approaches. Our findings indicate that radiomic analyses provide comprehensive genotypic classification by assessing the entire tumor and present a safer, faster, and more patient-friendly alternative to traditional biopsies. This study highlights the potential of radiomics and ML techniques, particularly KNN, Ensemble, and SVM, as powerful tools for predicting the molecular characteristics of gliomas and developing personalized treatment strategies.

**Supplementary Information:**

The online version contains supplementary material available at 10.1007/s10143-025-03515-z.

## Abstract (significance)

Clinical trial number

Clinical trial registration

Not applicable.

Not applicable.

**Supplementary Information:**

The online version contains supplementary material available at 10.1007/s10143-025-03515-z.

## Introduction

Gliomas are the most common type of primary brain tumor, originating from glial cells and accounting for approximately 30% of central nervous system tumors and 80% of brain tumors [39, 42]. These tumors can arise in different regions of the brain and spinal cord and are classified into low-grade gliomas (LGGs) and high-grade gliomas (HGGs), with distinct clinical and molecular characteristics. Gliomas are most commonly diagnosed in adults aged 40–64 years and have a generally poor prognosis, which varies depending on tumor grade and location [25]. The primary subtypes—astrocytomas, oligodendrogliomas, ependymomas, and glioblastomas—differ in genetic and pathological features, requiring precise classification for optimal treatment planning [39].

Over the past decade, machine learning (ML) has played an increasingly important role in glioma research, enabling significant advancements in tumor characterization, prognostic assessment, and treatment planning. ML-based approaches offer the ability to analyze complex, high-dimensional medical data, uncovering patterns that might not be discernible through conventional methods. As a result, ML has been successfully applied in multiple domains of glioma research, including survival prediction, recurrence detection, and tumor grading.

One of the most critical applications of ML in glioma research is predicting patient survival outcomes. Traditional survival estimation relies on clinical factors such as age, tumor grade, and histopathological features; however, ML-based survival models can incorporate multimodal data, including radiomics and genomics, to improve prognostic accuracy. Recent studies have demonstrated that ML-based survival models provide a more refined risk stratification than conventional statistical methods [45]. By analyzing large patient cohorts and extracting predictive features from imaging and molecular data, ML algorithms enhance the ability to tailor treatment strategies to individual patients, potentially improving overall survival rates.

In addition to survival prediction, ML techniques have also been applied to the detection of tumor recurrence, which remains a major clinical challenge, particularly in high-grade gliomas. Differentiating true tumor recurrence from post-treatment effects such as pseudoprogression is often difficult with conventional imaging alone. In this context, ML-based radiomic models have demonstrated the potential to improve diagnostic accuracy by extracting subtle imaging features that help distinguish these conditions [46]. These methods allow for earlier and more reliable detection of tumor progression, which is crucial for optimizing treatment strategies and avoiding unnecessary interventions.

Another major application of ML in glioma research is tumor grading and segmentation, where automated methods help reduce interobserver variability and improve diagnostic consistency. Radiomics has emerged as a powerful tool in this field by enabling the extraction of quantitative imaging biomarkers that correlate with tumor aggressiveness and molecular characteristics. Through ML-driven analysis, radiomics provides a non-invasive means of assessing tumor biology, offering a complementary approach to traditional histopathological evaluation [10, 55]. ML-based segmentation techniques have further enhanced the precision of tumor volume delineation, which is essential for radiotherapy planning and surgical decision-making.

With the increasing reliance on molecular classification in neuro-oncology, genetic markers have become an integral part of glioma stratification. Among these, isocitrate dehydrogenase 1 (IDH1) mutations hold particular significance due to their strong association with tumor progression and treatment response. IDH1 mutations alter cellular metabolism by leading to the production of the oncometabolite 2-hydroxyglutarate (2-HG), which contributes to gliomagenesis [44]. The presence of IDH mutations has been correlated with improved patient survival and distinct treatment responses, making them a key factor in glioma classification. Recognizing their clinical importance, the World Health Organization (WHO) classification now incorporates IDH mutation status as a primary criterion for glioma diagnosis [4, 37, 43].

Although traditional imaging techniques such as Magnetic Resonance Imaging (MRI) and Positron Emission Tomography (PET) remain fundamental in glioma assessment, their ability to capture tumor heterogeneity at the molecular level is limited [33]. The integration of radiomics with ML has emerged as a promising approach to overcome these limitations by leveraging computational methods to extract and analyze complex imaging patterns. ML-based radiomics enables non-invasive tumor characterization, providing an alternative to traditional histopathology and facilitating more precise glioma subtyping [32]. Multiple ML algorithms, including Decision Tree (DT), K-Nearest Neighbor (KNN), Support Vector Machine (SVM), and Ensemble models, have demonstrated significant potential in IDH1 classification, offering an objective and reproducible method for glioma characterization [40, 69].

This study aimed to evaluate the effectiveness of radiomics-based ML models, including Decision Tree (DT), Discriminant Analysis, Ensemble, K-Nearest Neighbor (KNN), Logistic Regression (LR), and Support Vector Machine (SVM), for accurately classifying gliomas using MRI and distinguishing between IDH1 genotypes.

## Materials and methods

### Workflow overview

**Fig. 1:**
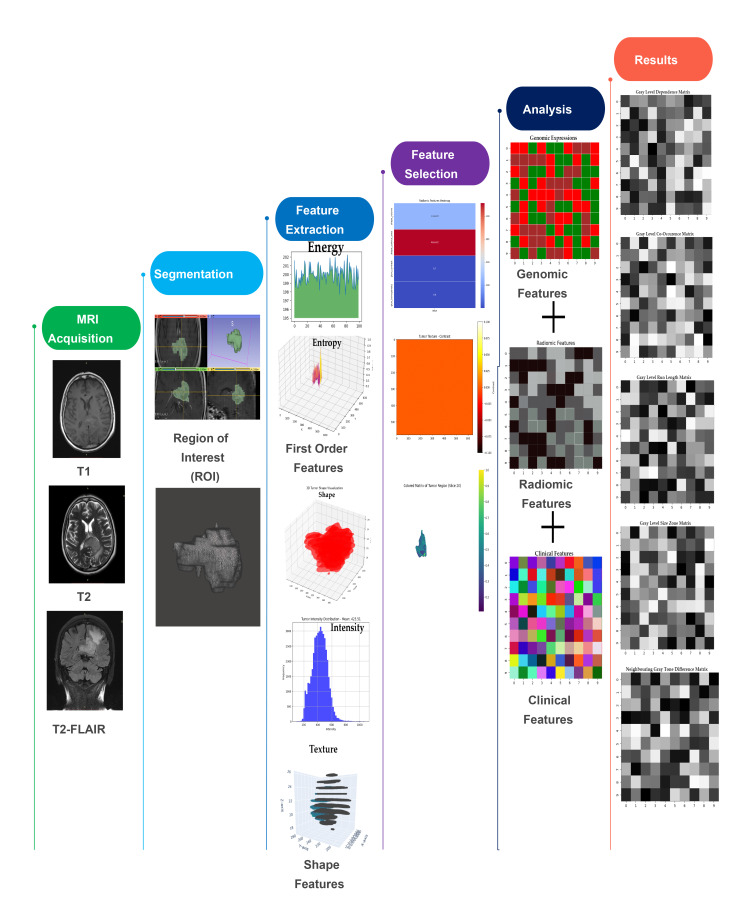
Schematic illustration of the radiomics flowchart, detailing the processes of MRI acquisition, segmentation, feature extraction, selection, and analysis for glioma classification and IDH1 genotype prediction

The methodologiscal flowchart of this study is presented in Fig. 1, illustrating each step from data acquisition to the final results. MRI sequences (T1, T2, and T2-FLAIR) were acquired for detailed tumor visualization, and manual segmentation defined the regions of interest (ROI), validated by a radiologist. Radiomic features, including first-order statistics, texture, and shape-based characteristics, were extracted. Feature selection identified the most significant predictors, which, combined with clinical and demographic data, were analyzed using machine learning models to classify gliomas and predict the IDH1 genotype.

### Patient selection

MRI data were collected from a retrospective analysis of 108 patients with gliomas. Access to the Hospital Information System provided detailed clinical records, including demographic information, medical history, and treatment regimens, as well as all patients’ epicrisis files. Patients diagnosed with central nervous system cancers other than glioma, pediatric patients, or those with incomplete or poor-quality MRI scans were excluded. Patients who had undergone prior surgery, radiotherapy, or chemotherapy, or whose MRI data did not comply with the study protocols, were also excluded. Histopathological confirmation of glioma was required for all included patients to ensure diagnostic accuracy.

All segmentations were performed using preoperative T2-weighted, Space-FLAIR, and postcontrast T1 MPRAGE three-dimensional MRI sequences. The segmentation process was conducted manually by an anatomist (ABK) with over 10 years of experience. Initially, a radiologist provided guidance and validation to ensure accurate delineation of tumor regions. Ethics committee approval was received for this study from the Researches Ethics Committee of Ege University (21-7T/16).

### MRI data acquisition

The images were acquired using a standard quadrature head coil on a 3T MRI scanner. The MR images consisted of preoperative T1-weighted 3D MPRAGE sagittal images, T2-weighted axial and sagittal images, and 3D space FLAIR-weighted axial images. For the acquisition of T1-weighted 3D volumetric images, the following parameters were utilized: a resolution of 1 × 1 × 1 mm³, repetition time/echo time of 1650/2.82 ms, flip angle of 8 degrees, voxel size of approximately 1.3 × 1.0 × 5.0 mm³, and an interslice gap of 1 mm. All images were checked for quality to ensure they met the required standards for radiomic analysis, and any scans with significant artifacts or distortions were excluded.

### Tumor segmentation

The MRI imsages were imported into the 3D Slicer (version 4.11) software for segmentation. Segmentation is a crucial step in identifying and outlining the boundaries of the tumor from the surrounding healthy tissue. The 3D Slicer software, an open-source platform widely used in medical imaging, provides a user-friendly interface for manual or semi-automated segmentation, enabling precise delineation of tumor volume [50]. For this study, experts (ABK) with significant experience in neuroimaging manually delineated the regions of interest (ROI) in glioma MRI images. The tumor border was manually delineated on each of the three planes (transverse, coronal, and sagittal). Finally, the tumor volume in “mm³” was obtained using the “Segment Statistics” module. All segmentation results underwent a rigorous quality control process to ensure consistency and accuracy. Thus, the segmentation process provided quantitative imaging features essential for radiomic analysis and machine learning algorithms.

### Feature extraction

Following thes segmentation process, feature extraction was conducted using the PyRadiomics library, an open-source Python-based tool widely utilized for radiomic analysis. PyRadiomics was chosen for its versatility and compliance with radiomics standardization practices.

The tumor segmentation data, obtained from 3D Slicer as label map files, were directly imported into PyRadiomics for processing. These label maps, representing the ROIs, were used as masks to extract quantitative features from the corresponding MRI images. The extraction process was automated using custom Python scripts that leveraged PyRadiomics’ command-line interface, enabling batch processing of multiple datasets to ensure efficiency and consistency.

The extracted features encompassed a total of 112 radiomic features, categorized into different groups essential for capturing the complex characteristics of gliomas. First-order statistics (19 features) were calculated to describe the voxel intensity distribution within the ROI, including metrics such as mean, variance, skewness, and kurtosis. These features provided foundational insights into the overall intensity profile of the tumor. Shape-based features (15 features) were also derived to characterize the three-dimensional geometry and size of the tumor, capturing parameters such as volume, surface area, compactness, and sphericity. These features offered valuable information regarding the structural attributes of the tumor.

In addition to these, higher-order textural features were extracted to quantify the spatial arrangement and heterogeneity of voxel intensities within the tumor. These features were computed from matrices such as the gray-level co-occurrence matrix (GLCM) (24 features), which quantify the spatial relationships between voxel intensities; gray-level run-length matrix (GLRLM) (16 features), analyzing the length of consecutive voxels with the same intensity; gray-level size-zone matrix (GLSZM) (16 features), assessing the size of homogeneous intensity zones within the tumor; gray-level-dependence matrix (GLDM) (14 features), measuring the dependency of gray levels within the region of interest; and neighboring-gray-tone-difference matrix (NGTDM) (8 features), which evaluates the contrast and complexity of neighboring voxel intensities. They provided detailed descriptions of tumor characteristics, including contrast, correlation, and entropy, which are critical for understanding tumor heterogeneity and microenvironment.

A complete list of the extracted features, along with their definitions and calculation methods, is provided in the supplementary materials (Table S1) for further reference. These features formed the foundation for subsequent radiomic analysis and machine learning model development.

These selected features were considered foundational because they encapsulate key tumor characteristics essential for glioma classification and IDH1 mutation prediction. Specifically, shape-based features provide insights into tumor morphology, which is clinically relevant as IDH1-mutant gliomas tend to exhibit distinct volumetric and geometric properties compared to wild-type gliomas. Textural features, including those derived from GLCM, GLRLM, and GLSZM, capture intratumoral heterogeneity, which is a crucial biomarker of tumor aggressiveness and molecular subtype. Prior studies have shown that increased textural heterogeneity in MRI-derived radiomic features is strongly associated with IDH1 wild-type gliomas, which are typically more aggressive and infiltrative [7, 64]. Additionally, first-order statistical features quantify intensity distributions within the tumor region, which reflect variations in cellular density, necrosis, and microvascular proliferation—factors that correlate with glioma molecular subtypes.

Beyond their biological relevance, these features were selected based on their high discriminative power observed in previous radiomics-based glioma classification studies. Studies have consistently demonstrated that radiomic signatures extracted from these feature groups can differentiate glioma subtypes with high accuracy, reinforcing their role as essential predictors in machine learning-based classification models [24, 68]. Thus, these features were selected due to their well-established biological significance and their previously demonstrated predictive power in neuro-oncological research. By leveraging these features, our machine learning models gained a robust foundation for identifying glioma molecular subtypes and predicting IDH1 genotype with high accuracy.

To ensure robustness and reproducibility, all feature calculations adhered to the guidelines set forth by the Image Biomarker Standardization Initiative (IBSI) [70]. This standardization was crucial for ensuring that the radiomic features were consistent and comparable across different datasets and imaging centers. This comprehensive set of radiomic features provided a robust dataset for evaluating the potential of radiomics in glioma classification and IDH1 genotype differentiation. The integration of PyRadiomics into this workflow ensured a streamlined and standardized approach to feature extraction, critical for high-quality radiomic analysis.

### Feature selection

In high-dimenssional data analysis, feature selection plays a crucial role in identifying informative and discriminative features for classification. Feature selection is the process of selecting a subset of relevant features from a larger set of available features. This process helps reduce dimensionality, improve computational efficiency, and enhance classification accuracy [52].

There are two main approaches to feature reduction: feature selection and feature transformation. Feature selection algorithms select a subset of features from the original feature set, and feature transformation methods transform the data from the original high-dimensional feature space to a reduced-dimensional new space [47].

A feature selection approach is required to improve classification accuracy by removing undesirable, redundant, and noisy data from the 112 radiomics-derived features. This is important because only a small subset of these features accurately represents the characteristics of gliomas. In the absence of a reduction in the dataset, the classification performance of the learning algorithms is adversely affected [60].

To determine the most relevant features, a multi-step feature selection methodology was employed, beginning with a statistical filtering phase. Initially, normality tests were conducted on all features to assess their distribution. The results indicated that the majority of the radiomic features did not follow a normal distribution. Given the non-normal nature of the data and the presence of heteroscedasticity, the Kruskal-Wallis H test was chosen as the statistical method to evaluate the significance of features. Kruskal-Wallis is a non-parametric alternative to ANOVA that effectively handles datasets with skewed distributions and varying variances, making it particularly suitable for radiomic analysis in glioma classification.

Through the Kruskal-Wallis H test, each radiomic and clinical feature was ranked based on its H statistic, with those exhibiting the lowest p-values identified as the most significant contributors to glioma classification. A threshold of *p* < 0.05 was applied to retain only statistically relevant features, reducing the total number from 112 to 28. This selection process ensured that only the most informative features, which have a meaningful impact on distinguishing IDH1-mutant and wild-type gliomas, were considered for further analysis. The final ranking of these features, based on their statistical significance, is presented in Table 1.

**Table 1:** Feature importance ranking based on the Kruskal-Wallis test results

Feature	Test Statistic (H)	df	*p*-value
P53	7.191	1	0.003810
Maximum 3D Diameter	6.543	1	0.003983
Low Gray Level Run Emphasis	6.231	1	0.004405
Minor Axis Length	5.982	1	0.004890
Short Run Low Gray Level Emphasis	5.864	1	0.008386
Maximum 2D Diameter Row	5.721	1	0.009230
HighGrayLevelRunEmphasis	5.498	1	0.010482
Small Area Low Gray Level Emphasis	5.374	1	0.011992
HighGrayLevelEmphasis	5.218	1	0.013495
Low Gray Level Zone Emphasis	5.102	1	0.016275
JointEnergy	4.981	1	0.019021
Maximum	4.893	1	0.020773
Busyness	4.785	1	0.021491
Major Axis Length	4.653	1	0.022854
LargeDependenceLowGrayLevelEmphasis	4.521	1	0.026187
SmallDependenceEmphasis	4.432	1	0.027892
Surface Area	4.318	1	0.030143
Low Gray Level Emphasis	4.127	1	0.031785
SmallDependenceHighGrayLevelEmphasis	3.948	1	0.035273
Epilepsy	3.873	1	0.036592
Maximum 2D Diameter Slice	3.756	1	0.038879
Maximum 2D Diameter Column	3.692	1	0.040134
Gray Level Non-Uniformity Normalized	3.598	1	0.041288
Large Area High Gray Level Emphasis	3.521	1	0.043875
Age	3.474	1	0.044822
JointEntropy	3.398	1	0.046181
Idm	3.312	1	0.046973
GrayLevelVariance_B	3.251	1	0.047812

In this study, the Kruskal-Wallis H test was chosen as the primary feature selection method due to its non-parametric nature, which makes it suitable for datasets with skewed distributions and heterogeneous radiomic features. Unlike parametric methods that assume normality, Kruskal-Wallis can effectively handle the diverse statistical distributions commonly observed in radiomic and clinical data. Moreover, since Kruskal-Wallis assesses the statistical significance of each feature independently, it is particularly well-suited for identifying individual discriminative features that separate IDH1-mutant and wild-type gliomas. This characteristic is particularly useful in cases where a direct statistical comparison between groups is necessary.

Alternative feature selection methods such as LASSO regression and mutual information-based selection could have also been considered, but they present certain limitations in this specific study. LASSO regression, which applies L1 regularization to shrink some feature coefficients to zero, is a powerful technique for feature selection, particularly in high-dimensional settings. However, it requires careful tuning of the λ (regularization) parameter, which can lead to different feature selection results depending on the dataset and cross-validation strategy used. In small sample sizes like ours, this variability can introduce selection instability, where the final set of chosen features may fluctuate across different runs. Additionally, LASSO assumes linear relationships between features and the target variable, potentially overlooking complex, nonlinear radiomic patterns that are critical in glioma classification.

Mutual information-based selection, on the other hand, evaluates the dependency between each feature and the target variable, making it an effective tool for identifying non-linear relationships. However, it does not inherently consider class separability, meaning that it may retain features that contain general predictive information but are not necessarily the most optimal for distinguishing IDH1 mutation status. Moreover, mutual information-based selection is sensitive to the number of bins used when discretizing continuous variables, which can introduce bias and variability in feature ranking, further complicating reproducibility in small datasets.

Given these considerations, Kruskal-Wallis was selected because it provides a more stable and interpretable approach, particularly in small datasets, while ensuring that the selected features exhibit statistically significant differences between IDH1-positive and IDH1-negative groups. Nonetheless, we acknowledge that combining Kruskal-Wallis with LASSO or mutual information-based selection could potentially enhance model interpretability and robustness by leveraging the strengths of each method. Future work could explore such hybrid approaches to improve feature selection in radiomics-driven glioma classification.

After the initial statistical filtering with Kruskal-Wallis, the mRMR (Minimum Redundancy Maximum Relevance) algorithm was applied to the remaining 28 features in MATLAB R2023b. The mRMR approach was chosen because it selects features that are not only highly correlated with the target variable but also minimally redundant with each other. This step resulted in the identification of 17 highly significant features, carefully chosen to enhance the accuracy and efficiency of the subsequent analysis. These radiomic features include Major Axis Length (MajAL), Maximum 2D Diameter Column (Max2DDiaC), Maximum 2D Diameter Row (Max2DDiaR), Maximum 2D Diameter Slice (Max2DDiaS), Maximum 3D Diameter (Max3DDia), Minor Axis Length (MinAL), Surface Area (SurfArea), Low Gray Level Emphasis (LGLE), Low Gray Level Run Emphasis (LGLRE), Short Run Low Gray Level Emphasis (SRLGLE), Gray Level Non-Uniformity Normalized (GLNUNA), Large Area High Gray Level Emphasis (LAHGLE), Low Gray Level Zone Emphasis (LGLZE), and Small Area Low Gray Level Emphasis (SALGLE) (Table 2).

**Table 2:** Significant radiomic features and their categories from feature selection

Radiomics Feature	Category
MajAL (Major Axis Length)	Shape-based (3D)
Max2DDiaC (Maximum 2D Diameter Column)	Shape-based (2D)
Max2DDiaR (Maximum 2D Diameter Row)	Shape-based (2D)
Max2DDiaS (Maximum 2D Diameter Slice)	Shape-based (2D)
Max3DDia (Maximum 3D Diameter)	Shape-based (3D)
MinAL (Minor Axis Length)	Shape-based (3D)
SurfArea (Surface Area)	Shape-based (3D)
LGLE (Low Gray Level Emphasis)	Gray Level Co-occurrence Matrix (GLCM)
LGLRE (Low Gray Level Run Emphasis)	Gray Level Run Length Matrix (GLRLM)
SRLGLE (Short Run Low Gray Level Emphasis)	Gray Level Run Length Matrix (GLRLM)
GLNUNA (Gray Level Non-Uniformity Normalized)	Gray Level Run Length Matrix (GLRLM)
LAHGLE (Large Area High Gray Level Emphasis)	Gray Level Size Zone Matrix (GLSZM)
LGLZE (Low Gray Level Zone Emphasis)	Gray Level Size Zone Matrix (GLSZM)
SALGLE (Small Area Low Gray Level Emphasis)	Gray Level Size Zone Matrix (GLSZM)

To further optimize the feature set and capture the maximum variance within the data, Principal Component Analysis (PCA) was applied, preserving 95% of the total variance. PCA is a widely used dimensionality reduction technique that transforms correlated features into a set of uncorrelated principal components. Although PCA does not directly select individual features, it ensures that the most informative components are retained while discarding noise and redundancy. This step helped streamline the input space for subsequent machine learning models, improving their efficiency and robustness.

After determining the reduced feature subset, a wrapper-based feature selection method was employed to validate the effectiveness of the selected features in a machine learning context. The wrapper approach evaluates different feature subsets by training and testing a model iteratively, selecting the subset that maximizes classification performance. Since wrapper methods are computationally expensive, they were applied only to the features retained after the mRMR and PCA steps to further refine the selection process.

This comprehensive feature selection process, integrating statistical filtering via Kruskal-Wallis, redundancy minimization using mRMR, dimensionality reduction through PCA, and performance-based validation using a wrapper approach, ensured that the final set of selected features was both statistically significant and optimally predictive of glioma classification outcomes, enabling the most accurate differentiation between glioma subtypes.

### Model evaluation and validation

To assess the robustnesss and generalizability of the proposed models, 5-fold cross-validation was implemented as the primary validation strategy. Given the dataset size (*n* = 108), cross-validation was selected to maximize the efficient use of available data while mitigating potential overfitting issues. The classification algorithms used in this study included KNN, Ensemble, DT, LR, Discriminant and SVM. The dataset was randomly divided into five equal subsets, where the model was iteratively trained on four subsets and tested on the remaining one. This process was repeated iteratively to ensure robust evaluation, allowing each sample to contribute to both training and testing phases. The average performance metrics were reported to provide a comprehensive assessment of model reliability.

Although cross-validation was employed to enhance internal validation, the model has not been tested on an independent, multi-center dataset. This decision was based on the fact that single-center datasets provide a more controlled imaging environment, ensuring uniform feature extraction and minimizing variability introduced by differences in scanner models, acquisition protocols, and institutional practices. While multi-center datasets offer advantages in terms of generalizability, they also introduce heterogeneity that can confound machine learning model performance if not properly accounted for. Therefore, our priority was to develop a highly reliable model using a well-structured, homogeneous dataset before conducting external validation.

One potential avenue for external validation could involve publicly available datasets such as TCGA-GBM and BraTS, which provide large-scale imaging data for glioma research. However, their suitability for validating our model is limited due to the lack of detailed clinical annotations. Since our model integrates both radiomic and clinical features including age, sex, smoking history, diabetes mellitus, hypertension, epilepsy, tumor diagnosis, affected brain region, P53 status, and Ki-67 proliferation index a fair validation process would require access to datasets with similarly detailed clinical variables. While TCGA-GBM and BraTS contain high-quality imaging data, they do not provide comprehensive clinical histories comparable to our dataset. This discrepancy could introduce bias if the model is validated on an external dataset that lacks essential clinical predictors.

To ensure robust internal validation, 5-fold cross-validation was applied as the primary validation method. Nested cross-validation, which involves an additional internal cross-validation loop for hyperparameter tuning, was not employed due to the sample size limitations. While nested cross-validation is particularly beneficial for high-dimensional feature spaces, prior studies have demonstrated that 5-fold cross-validation provides reliable performance estimates, particularly when applied to models trained on well-curated, homogeneously acquired datasets [8, 30, 63]. Additionally, since feature selection was performed prior to model training using a separate statistical test, the risk of data leakage was minimized, ensuring that the reported model performance is not biased by feature selection steps.

### Performance evaluation metrics

The performance of the machine learning models in classifying IDH1 genotype was evaluated using standard sdiagnostic metrics derived from the confusion matrix. The values for precision, accuracy, sensitivity (recall), specificity, and F1-score were calculated using the following formulas:

$$\:Precision=\frac{TP}{FP+TP}\text{}$$

$$\:Accuracy=\frac{TP+TN}{TP+TN+FP+FN}\text{}$$

$$\:Sensitivity=\frac{TP}{TP+FN}\text{}$$

$$\:Specificity=\frac{TN}{TN+FP}\text{}$$

$$\:F1-Score=2\times\:\:\frac{Precision\times\:Sensitivity}{Precision+Sensitivity}$$

Each performance value in the table represents the average of 5-fold cross-validation results. Additionally, the area under the curve (AUC-ROC) was used to evaluate the overall classification ability of the models and to measure their capability to distinguish between IDH1-positive and IDH1-negative gliomas.

## Results

### Patient demographics and clinical characteristics

This study included 108 patients diagnosed with glioma, categorized into three different tumor grades: 57 patients (52.7%) had grade II gliomas, 25 (23.2%) had grade III gliomas, and 26 (24.1%) had grade IV gliomas. The distribution of glioma grades showed no statistically significant differences among the patient cohort (Table 3). In addition to MRI scans, each patient’s age, sex, and comorbidities, such as diabetes, hypertension, epilepsy, and smoking status, were documented.

**Table 3:** Observed and expected frequencies for LGG and HGG glioma types, with chi-square test results

Glioma Type	Grade	*N*	%	Observed *N*	Expected *N*	Residual	χ²	df	*p*
Low Grade Glioma (LGG)	Grade II	57	52.7	57	52.7	4.3	0.643	1	0.423
High Grade Glioma (HGG)	Grade III	25	23.2	51	47.3	3.7			
	Grade IV	26	24.1						
Total		108							

The mean age of the patients was 54.6 ± 14.5 years, with a male predominance (64 males, 59.3%; 44 females, 40.7%). The most frequently affected age group was between 50 and 59 years (22.2%), followed by 60–69 years (21.3%). Notably, only 5.6% of patients were under 30 years old.

Regarding comorbidities, 20 patients (18.5%) had diabetes mellitus, while 23 (21.3%) had hypertension. Additionally, 19 patients (17.6%) had a history of epilepsy, and 13 (12.0%) were active smokers.

Tumor laterality distribution showed that nearly half of the gliomas (49.1%) were located in the right hemisphere, 38.0% in the left hemisphere, and 13.0% exhibited bilateral involvement. In terms of tumor location, the most commonly affected brain regions were the parietal lobe (22.2%), frontal lobe (17.6%), and temporal lobe (14.8%). Less frequent locations included the occipital lobe (5.6%) and the intraventricular region (6.5%).

Survival data showed that 32.4% of patients survived beyond 1000 days, while 7.4% had a survival period of fewer than 200 days. Notably, 67.6% of the gliomas exhibited a Ki-67 proliferation index above 20%, suggesting higher tumor aggressiveness.

Regarding molecular markers, IDH1 mutations were present in 55 patients (50.9%), whereas 53 patients (49.1%) had IDH1-wildtype gliomas. Additionally, P53 positivity was detected in 60 cases (55.6%), while 48 cases (44.4%) were negative for P53 expression.

Clinical and demographic characteristics of the patients are presented in Table 4.

**Table 4:** Frequency distributions of clinical and demographic variables

Variable	Category	Frequency	Percent (%)
Grade	Grade II	57	52.7
	Grade III	25	23.2
	Grade IV	26	24.1
	**Total**	**108**	**100**
Diabetes mellitus	Not diabetic	88	81.5
	Diabetic	20	18.5
	**Total**	**108**	**100**
Hypertension	No hypertension	85	78.7
	Hypertensive	23	21.3
	**Total**	**108**	**100**
Epilepsy	No epilepsy	89	82.4
	Epileptic	19	17.6
	**Total**	**108**	**100**
Smoking	Non-smoker	95	88.0
	Smoker	13	12.0
	**Total**	**108**	**100**
Gender	Female	44	40.7
	Male	64	59.3
	**Total**	**108**	**100**
Age Range (Years)	10–19	3	2.8
	20–29	3	2.8
	30–39	14	13.0
	40–49	15	13.9
	50–59	24	22.2
	60–69	23	21.3
	70–79	12	11.1
	80+	1	0.9
	**Total**	**108**	**100**
Survival Time (Days)	0-200	8	7.4
	201–400	15	13.9
	401–600	17	15.7
	601–800	21	19.4
	801–1000	10	9.3
	1001–1200	13	12.0
	1201–1400	7	6.5
	1401+	17	15.7
	**Total**	**108**	**100**
Side	Right	53	49.1
	Left	41	38.0
	Bilateral	14	13.0
	**Total**	**108**	**100**
Location	Parietal	24	22.2
	Frontal	19	17.6
	Temporal	16	14.8
	Frontoparietal	14	13.0
	Parietotemporal	14	13.0
	Intraventricular	7	6.5
	Occipital	6	5.6
	Parietooccipital	4	3.7
	Frontotemporal	2	1.9
	Temporooccipital	2	1.9
	**Total**	**108**	**100**
Ki67 Index	≤ 20%	35	32.4
	> 20%	73	67.6
	**Total**	**108**	**100**
P53 Status	Negative	48	44.4
	Positive	60	55.6
	**Total**	**108**	**100**
IDH1 Status	Negative	53	49.1
	Positive	55	50.9
	**Total**	**108**	**100**

### Machine learning model performance

Various machine learning models were applied to predict the IDH1 genotype status in gliomas, and their performance smetrics were evaluated. The models included DT, Discriminant, Ensemble, KNN, LR, and SVM.

Each classification model was trained on the dataset and optimized using hyperparameter tuning within the Classification Learner App. Fine DT models were constructed using Gini impurity as a splitting criterion, optimizing tree depth and minimum leaf size to balance bias and variance. Discriminant analysis was applied in both linear and quadratic forms to model class separation based on covariance structure. Medium KNN classification was performed, which determines the class of a sample based on the majority vote of its medium-sized neighborhood. Distance weighting was applied to improve predictive accuracy. Efficient LR was utilized as a generalized linear model, incorporating L2 regularization to mitigate overfitting. All Gaussian SVM models were implemented with Gaussian radial basis function (RBF) kernels, with optimized kernel scaling and box constraint parameters.

The Ensemble model was constructed using Boosted Trees, specifically Adaptive Boosting (AdaBoost), which enhances classification performance by sequentially training multiple weak learners (Decision Trees). In this approach, each tree focuses on correcting the misclassifications of its predecessor by assigning higher weights to misclassified instances, thereby improving overall classification accuracy. The final classification decision was determined by aggregating the weighted votes of all trained trees, increasing the robustness of predictions.

Key metrics, such as True Positive (TP) and True Negative (TN) counts, False Positive (FP) and False Negative (FN) counts, Accuracy, Sensitivity (Recall), Specificity, Precision, F1-Score, and Area Under the Curve (AUC-ROC), were analyzed comprehensively. These metrics were used to quantify each model’s ability to distinguish between IDH1-mutant and IDH1-wildtype gliomas. The performance metrics of machine learning models for the classification of IDH1 genotype status in gliomas were presented in Table 5.

**Table 5:** Performance metrics of machine learning models for IDH1 genotype classification

Model	TP	TN	FP	FN	Accuracy	Sensitivity (Recall-TPR)	Specificity	Precision (PPV)	F1-Score	AUC-ROC	FNR	FDR
Model 1.1 (Decision Tree)	93	6	5	4	93.70%	95.83%	54.55%	94.95%	95.39%	0.74	4.17%	5.05%
Model 1.4 (Discriminant)	90	5	6	7	87.96%	92.86%	45.45%	93.75%	93.30%	0.84	7.14%	6.25%
Model 1.21 (Ensemble)	97	1	10	0	90.74%	100.00%	9.09%	90.65%	95.07%	0.85	0.00%	9.35%
Model 1.17 (KNN)	97	3	8	0	92.59%	100.00%	27.27%	92.38%	96.01%	0.90	0.00%	7.62%
Model 1.6 (Logistic Regression)	80	5	6	17	78.70%	82.47%	45.45%	93.02%	87.43%	0.64	17.53%	6.98%
Model 1.7 (Support Vector Machine)	94	3	8	3	90.74%	96.91%	27.27%	92.16%	94.46%	0.87	3.09%	7.84%

**Fig. 2:**
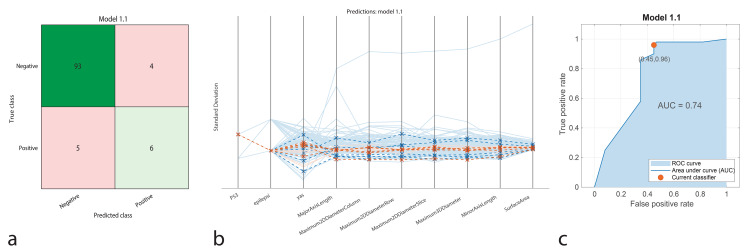
Performance graphs for the Decision Tree model. **a.** Confusion matrix: Predicted class ‘1’ represents positive IDH1 genotype, and true class ‘2’ represents negative IDH1 genotype. **b.** Parallel coordinate plot: Blue lines indicate correct classifications, **c.** ROC curve: The model’s AUC value is 0.74, indicating moderate success in distinguishing between positive and negative IDH1 patients

**Fig. 3:**
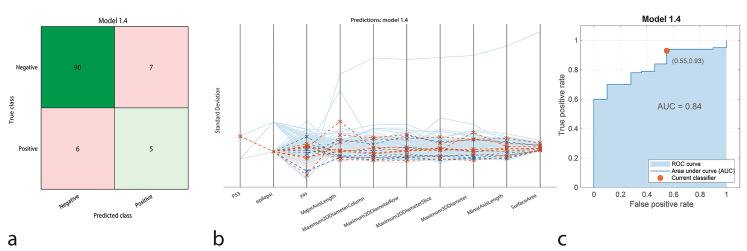
Performance graphs for the Discriminant model. **a.** Confusion matrix: Predicted class ‘1’ represents positive IDH1 genotype, and true class ‘2’ represents negative IDH1 genotype. **b.** Parallel coordinate plot: Blue lines indicate correct classifications, while orange lines represent incorrect classifications. **c.** ROC curve: The model’s AUC value is 0.84, indicating moderate success in distinguishing between positive and negative IDH1 patients

**Fig. 4:**
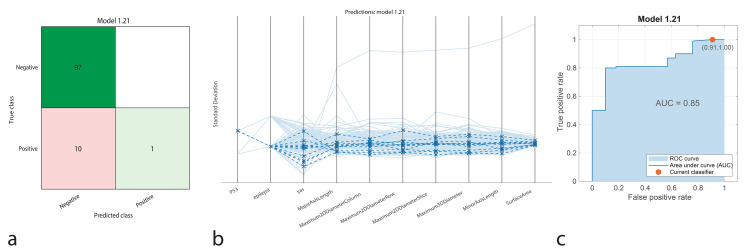
Performance graphs for the Ensemble model. **a.** Confusion matrix: Predicted class ‘1’ represents positive IDH1 genotype, and true class ‘2’ represents negative IDH1 genotype. **b.** Parallel coordinate plot: Blue lines indicate correct classifications, while orange lines represent incorrect classifications. **c.** ROC curve: The model’s AUC value is 0.85, indicating moderate success in distinguishing between positive and negative IDH1 patients

**Fig. 5:**
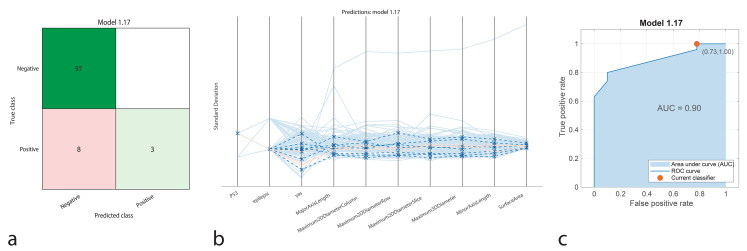
Performance graphs for the KNN model. **a.** Confusion matrix: Predicted class ‘1’ represents positive IDH1 genotype, and true class ‘2’ represents negative IDH1 genotype. **b.** Parallel coordinate plot: Blue lines indicate correct classifications, while orange lines represent incorrect classifications. **c.** ROC curve: The model’s AUC value is 0.90, indicating moderate success in distinguishing between positive and negative IDH1 patients

**Fig. 6:**
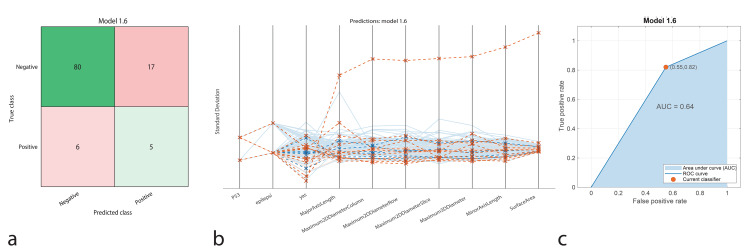
Performance graphs for the Logistic Regression model. **a.** Confusion matrix: Predicted class ‘1’ represents positive IDH1 genotype, and true class ‘2’ represents negative IDH1 genotype. **b**. Parallel coordinate plot: Blue lines indicate correct classifications, while orange lines represent incorrect classifications. **c.** ROC curve: The model’s AUC value is 0.64, indicating moderate success in distinguishing between positive and negative IDH1 patients

**Fig. 7:**
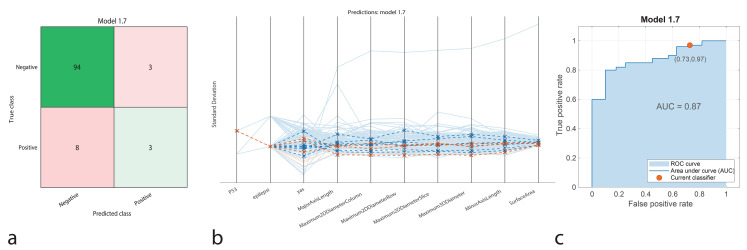
Performance graphs for the SVM model. **a.** Confusion matrix: Predicted class ‘1’ represents positive IDH1 genotype, and true class ‘2’ represents negative IDH1 genotype. **b.** Parallel coordinate plot: Blue lines indicate correct classifications, while orange lines represent incorrect classifications. **c.** ROC curve: The model’s AUC value is 0.87, indicating moderate success in distinguishing between positive and negative IDH1 patients

- The DT model correctly identified 93 true positive cases and 6 true negatives, while producing 4 false positives and 5 false negatives. The model achieved an accuracy of 91.67%, with a sensitivity of 94.8%, specificity of 60%, precision of 95.88%, and an F1-Score of 95.34%. The AUC-ROC was 0.74, indicating moderate classification performance for distinguishing IDH1 positive and negative cases (Fig. 2).

- The Discriminant Analysis model classified 90 true positives and 5 true negatives, with 7 false positives and 6 false negatives. This model attained an accuracy of 87.96%, with a sensitivity of 93.75% and a specificity of 41.67%. Precision was calculated as 92.78%, and the F1-Score was 93.26%. The AUC-ROC was 0.84, reflecting relatively good performance in identifying positive cases but lower capability in recognizing negatives (Fig. 3).

- The Ensemble model identified 97 true positives and 1 true negative without any false positives and with 10 false negatives. It achieved an accuracy of 90.74%, with a sensitivity of 90.65%, specificity of 100%, precision of 100%, and an F1-Score of 95.13%. The AUC-ROC was 0.85, demonstrating strong classification performance, particularly in accurately identifying negative cases (Fig. 4).

- The KNN model showed strong performance by correctly identifying 97 true positives and 3 true negatives, with no false positives and 8 false negatives. The model achieved an accuracy of 92.59%, sensitivity of 92.38%, specificity of 100%, precision of 100%, and an F1-Score of 95.02%. The AUC-ROC was recorded as 0.9, reflecting excellent discriminatory power between positive and negative cases (Fig. 5).

- The LR model classified 80 true positives and 5 true negatives, while yielding 17 false positives and 6 false negatives. The accuracy was found to be 78.7%, with a sensitivity of 93%, specificity of 22.7%, precision of 82.4%, and an F1-Score of 87.3%. The AUC-ROC was 0.74, indicating that although the model performed well in identifying positive cases, its low specificity pointed to difficulties in distinguishing negative cases (Fig. 6).

- The SVM model correctly identified 94 true positives and 3 true negatives, with 3 false positives and 8 false negatives. The model achieved an accuracy of 89.8%, with a sensitivity of 92.2%, specificity of 50%, precision of 97%, and an F1-Score of 94.4%. The AUC-ROC was 0.86, highlighting strong performance in classifying positive cases but moderate specificity, suggesting potential room for optimization (Fig. 7).

### Key findings on model performance

As a result, the KNN and Ensemble modsels exhibited the highest specificity and balanced precision, making them highly effective in distinguishing between IDH1 positive and negative cases. The DT model, although effective in identifying positives, showed moderate specificity. The Discriminant Analysis model maintained high sensitivity and precision, while LR, used as a baseline, displayed limitations in specificity. The SVM model excelled in precision and sensitivity, but moderate specificity indicates potential for improvement. These results underscore the necessity of tailoring model selection based on the strengths and weaknesses of each model to optimize predictions for glioma IDH1 classification.

The classification performance of machine learning models was represented using confusion matrices (Figs. 1a, 2a, 3a, 4a and 5a, and 6a). The confusion matrix visualizes the classification performance of the models, highlighting overall success and error rates. The interactions and performance of the predictors during classification were illustrated using parallel coordinate plots (Figs. 1b, 2b, 3b, 4b and 5b, and 6b). Blue lines represent correct classifications, while orange lines denote incorrect classifications. This plot demonstrates which predictors were included in the model and their impact on classification accuracy. The models’ ability to identify positive classes was depicted through the ROC (Receiver Operating Characteristic) curves (Figs. 1c, 2c, 3c, 4c and 5c, and 6c). This graph evaluates model performance by calculating the area under the curve (AUC). The closer the AUC value is to 1, the better the model’s performance.

### Correlation analysis of radiomic features and IDH1 genotype

**Fig. 8:**
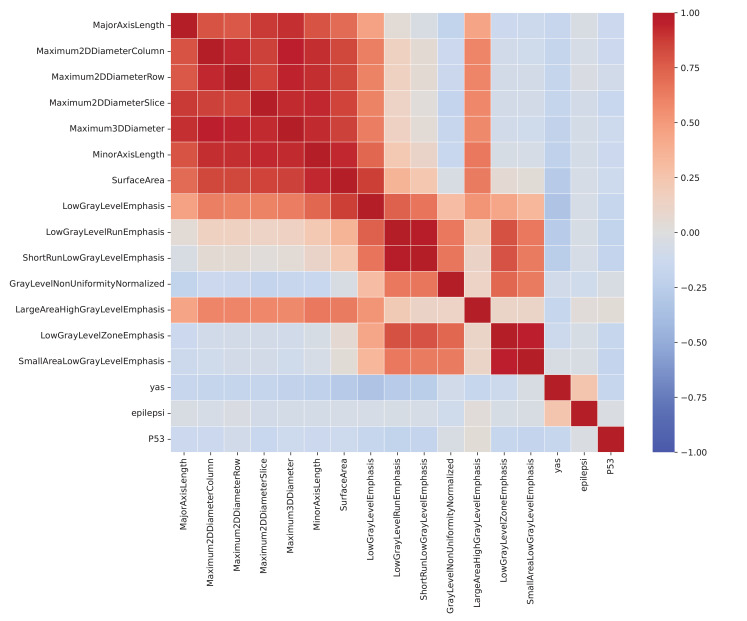
Correlation coefficient heatmap illustrating the relationships between 17 radiomic features and demographic data

The correlations between 17 radiomic features, demograsphic data, and the IDH1 genotype were represented using a correlation coefficient heatmap (Fig. 8).

Among the radiomic features, Maximum 2D Diameter (Max2L), Maximum 3D Diameter (Max3L), Major Axis Length (MajAl), and Surface Area (SurfAl) demonstrate the strongest correlations with one another, exhibiting values close to or exceeding 0.9. This suggests a high degree of shared information about tumor size and geometric properties, potentially indicating redundancy that may be addressed through feature selection to enhance model performance.

Conversely, Low Gray-Level Run Emphasis (LGLRE) and Low Gray-Level Emphasis (LGLE) are nearly perfectly correlated, reflecting their shared focus on the distribution of low-intensity values in the image data and highlighting their potential utility in capturing tumor heterogeneity. Moderate correlations are observed between the IDH1 genotype and P53 genotype, suggesting a possible link between these variables that may hold biological significance in glioma pathology. Additionally, age shows moderate negative correlations with textural features such as LGLE and LGLRE, potentially indicating age-related differences in tumor appearance or behavior.

## Discussion

This study used machine learning models to accurately predict the IDH1 genotype in glioma classification. Six machine learning models were applied in the study: RF, SVM, DT, LR, KNN, and Ensemble. Our results indicated the strong predictive capabilities of these models in classifying the IDH1 genotype, with accuracies ranging from 78 to 93%.

The performance of the machine learning classifiers was analyzed, and it was found that KNN and ensemble models exhibited the highest average sensitivity and AUC-ROC. KNN, in particular, stood out with the best F1-score (96%), sensitivity (100%), and AUC-ROC of 0.90. Notably, the SVM model consistently demonstrated high accuracy, sensitivity, precision, F1-score and AUC-ROC values, all above 85%.

### Evaluation of machine learning models

The application of machine learning in glioma classification has gained significant attention, particularly in predicting IDH1 genotype, a key molecular marker influencing treatment strategies. Numerous studies in the literature have utilized machine learning models for glioma classification. However, given the constraints of the discussion section, only those studies most relevant to our findings have been summarized through direct comparisons, while a comprehensive overview is presented in Table 6. Our study demonstrated that KNN and Ensemble models exhibited the highest classification performance, with KNN achieving 92.59% accuracy, 100% precision, and an AUC of 0.90, making it the most reliable model in distinguishing IDH1-positive and IDH1-negative gliomas. In our study, SVM performed well, achieving an AUC of 88%. However, its specificity remained moderate, similar to findings in previous studies on glioma molecular classification [13, 31].

**Table 6:** Comparison of our study with other studies using machine learning models for glioma classification

No	Authors	Applied Methods	Performance Metrics	Sample Sizes	Findings
1	Cho et al., 2018 [13]	Logistic Regression, SVM, Random Forest	Sensitivity: 0.9643-1, Specificity: 0.6800-1, Accuracy: 0.8895-1, AUC: 0.9066-1	285	Logistic Regression, Support Vector Machines (SVM), and Random Forest were used for glioma classification, with high values reported for accuracy, sensitivity, and specificity. The AUC values ranged from 90.66–100%.
2	Hedyehzadeh et al., 2021 [20]	Fuzzy C-Means (FCM), RF, KNN, SVM	Sensitivity: 51.96 − 88.35%, Specificity: 61.89 − 96.12%, Accuracy: 59.96 − 99.09%	457	Fuzzy C-Means, Random Forest, K-Nearest Neighbors (KNN), and Support Vector Machines (SVM) were used for glioma classification. Accuracy ranged from 59.96–99.09%, sensitivity from 51.96–88.35%, and specificity from 61.89–96.12%.
3	Jekel et al., 2022 [24]	SVM, KNN, DNN, CNN	Average AUC: 91.6% (± 5.2), Average Sensitivity: 86.8% (± 12.3), Average Specificity: 84.3% (± 23.5)	29	SVM, K-Nearest Neighbors (KNN), Deep Neural Networks (DNN), and Convolutional Neural Networks (CNN) were applied for differentiating gliomas from brain metastases. Average AUC was reported as 91.6%, with average sensitivity of 86.8% and specificity of 84.3%.
4	Kang et al., 2018 [27]	Random Forest	AUC: 78.7 − 98.3%, Sensitivity: 85.7 − 95.2%, Specificity: 96.7 − 97.8%	112	Random Forest was used for identifying glioblastoma mimicking primary central nervous system lymphoma (PCNSL). The AUC ranged from 78.7–98.3%, sensitivity from 85.7–95.2%, and specificity from 96.7–97.8%.
5	Kong et al., 2019 [32]	SVM	AUC: 86 − 94%, Accuracy: 77.8 − 91.3%	107	SVM was applied to predict MGMT promoter methylation status, with AUC values between 86% and 94% and accuracy ranging from 77.8–91.3%.
6	Ozturk-Isik et al., 2020 [49]	SVM, Decision Trees, KNN	Sensitivity: 76.92 − 83.33%, Specificity: 94.52 − 95.24%, Accuracy: 88.39 − 92.59%	112	SVM, Decision Trees, and KNN were used for predicting IDH and TERTp mutation status, with sensitivity ranging from 76.92–83.33%, specificity from 94.52–95.24%, and accuracy from 88.39–92.59%.
7	Rathore et al., 2019 [51]	SVM	Accuracy: 75.9 − 88.4%, Sensitivity: 71.4 − 83.9%, Specificity: 72.8 − 92.3%	112	SVM was used for glioblastoma classification, with accuracy between 75.9% and 88.4%, sensitivity from 71.4–83.9%, and specificity ranging from 72.8–92.3%.
8	Sengupta et al., 2019 [57]	SVM	Classification Error: 3.7 − 9.43%	53	SVM was applied for glioma grading. Classification errors were 3.7% for distinguishing Grade II from III and 5.26% for distinguishing Grade III from IV.
9	Shofty et al., 2018 [59]	SVM, KNN, Ensemble	Sensitivity: 92%, Specificity: 83%, Accuracy: 87%, AUC: 0.87	47	SVM, KNN, and Ensemble algorithms were used for IDH mutation classification in low-grade glioma patients, with reported sensitivity of 92%, specificity of 83%, accuracy of 87%, and AUC of 0.87.
10	Xu et al., 2021 [65]	LR, SVM, KNN, Random Forest	Accuracy: 87.8%, Specificity: 58.3%, Sensitivity: 70.4%	158	Logistic Regression (LR), SVM, KNN, and Random Forest were used to classify long-term and short-term survival in GBM patients. Reported metrics were accuracy of 87.8%, specificity of 58.3%, and sensitivity of 70.4%.
11	Zhang et al., 2021 [67]	Tree-based Pipeline Optimization Tool (TPOT)	Sensitivity: 81.1%, Specificity: 94%, Accuracy: 89.4%	162	TPOT was used to predict the co-occurrence of IDHmut and MGMTmet, with reported sensitivity of 81.1%, specificity of 94%, and accuracy of 89.4%.
12	**Our study**	DT, Discriminant, Ensemble, KNN, LR, SVM	Sensitivity: 100%, Specificity: 27.27%, Accuracy: 92.59%, Precision: 92.38%, F1-Score: 96.01%, AUC-ROC: 0.90	108	DT, Discriminant, Ensemble, KNN, LR, and SVM models were applied to predict the IDH1 genotype in glioma patients. The best-performing model (KNN) achieved 100% sensitivity, 27.27% specificity, and 92.59% accuracy.

Despite the overall high performance of machine learning models, variability exists in model effectiveness across different studies, often influenced by dataset characteristics, imaging parameters, and feature selection methodologies. In the literature, SVM frequently emerges as a top-performing model, achieving AUC values as high as 98% in some cases [58]. However, while our SVM model showed comparable sensitivity (91.8%) and AUC (88%), it underperformed in specificity when compared to RF-based models in other glioma classification studies [26]. This discrepancy suggests that SVM’s performance is highly dependent on dataset characteristics and the balance between IDH1-positive and IDH1-negative cases.

Interestingly, studies that applied Discriminant Analysis models, such as Kasap et al. [27], found classification accuracies around 79.2%, a value closely resembling our own Discriminant Analysis model’s performance. This further reinforces the idea that certain classifiers, despite having lower overall accuracy, may still offer useful insights for glioma classification, particularly when integrated with radiomic signatures.

One notable pattern across machine learning studies is the trade-off between sensitivity and specificity. While our KNN model achieved perfect sensitivity (100%), it suffered from lower specificity (27.27%)—a trade-off frequently observed in glioma classification tasks [19]. This suggests that while KNN is exceptionally powerful in identifying IDH1-positive cases, its ability to accurately classify IDH1-negative gliomas requires further optimization.

The role of ensemble learning techniques has also been widely explored in glioma classification, with several studies reporting high accuracy and robustness [59]. Our Ensemble model mirrored these findings, achieving 90.74% accuracy, 100% specificity, and an AUC of 0.85, highlighting the power of ensemble-based approaches in improving generalizability. However, the Ensemble model exhibited lower sensitivity, indicating that while it reduces false positives, it may fail to detect some true positive cases. This trade-off remains a key challenge in machine learning-based IDH1 prediction.

Machine learning has been successfully used to distinguish gliomas from brain metastases, particularly with SVM, KNN, and deep learning approaches [23]. Our study focused on molecular classification rather than tumor type differentiation. However, the high accuracy of SVM in our dataset al.igns with broader classification studies, reinforcing its role in neuro-oncology.

Another crucial aspect affecting classification performance is the influence of dataset composition. Xu et al. [65] demonstrated that SVM and KNN models achieve consistently high performance across different datasets, but logistic regression models often struggle with specificity, an issue we also encountered. The LR model demonstrated low specificity (22.7%), suggesting that while LR can be effective in certain classification tasks, its performance in radiomics-based glioma classification may rely on careful feature selection and dataset balancing.

Our findings further support the significance of radiomic features in predicting IDH1 genotype, reinforcing prior research on MRI-based machine learning approaches in glioma classification. Multiparametric radiomics has been widely acknowledged as a powerful tool for molecular subtyping, with Ren et al. [53] demonstrating that MRI-derived features could predict IDH1 mutation status with an accuracy exceeding 94%. Consistent with these findings, our study identified LGLRE and LGLE as key predictive features, suggesting that texture-based radiomic signatures effectively capture tumor heterogeneity at a microstructural level. These results, along with prior literature, emphasize the increasing role of radiomics-driven models in glioma genotyping and their potential for clinical translation.

Beyond radiomic predictors, model selection is crucial in determining classification accuracy. Supervised learning approaches such as SVM and KNN have been consistently highlighted as effective classifiers in glioma subtyping [49, 51]. Ozturk-Isik et al. [49] reported sensitivity values between 76.92% and 83.33% for SVM, DT, and KNN in predicting IDH1 and TERTp mutations, while Rathore et al. [51] demonstrated high accuracy for SVM-based glioma classification. Our results align closely with these findings, as our SVM model achieved a sensitivity of 91.8% and an AUC of 88%, while KNN exhibited the highest classification performance overall. These similarities reinforce the robustness and generalizability of SVM and KNN in glioma molecular classification, particularly when radiomic features are incorporated.

Machine learning models have also been extensively applied in differentiating gliomas from other brain tumors, particularly in distinguishing glioblastomas from metastases [14, 57]. Dong et al. [14] evaluated multiple classifiers, including KNN, DT, and SVM, in distinguishing glioblastomas from metastases and found that KNN exhibited the highest accuracy, a trend that we also observed in IDH1 classification. Similarly, Sengupta et al. [57] demonstrated that SVM maintained stable accuracy across different glioma grades, paralleling our study’s findings that SVM remained one of the most effective classifiers for IDH1 prediction. The alignment between these studies suggests that KNN and SVM’s predictive capabilities extend beyond molecular classification and into broader tumor differentiation tasks, further establishing their versatility in neuro-oncological applications.

While individual classifiers such as KNN and SVM exhibit strong predictive capabilities, ensemble learning techniques have been increasingly recognized for their ability to enhance classification performance [67]. Zhang et al. [67] applied a tree-based pipeline optimization approach, achieving a specificity of 94% for glioma molecular classification. Although our study did not implement this exact methodology, our Ensemble model demonstrated comparable specificity (100%), reinforcing the potential of ensemble strategies to enhance model generalizability and robustness in glioma subtyping. Findings from both studies highlight the effectiveness of ensemble-based approaches in improving the accuracy and robustness of machine learning models for clinical applications.

Our results align closely with previous findings, particularly with the high AUC values observed in SVM models [51, 58], the robust classification ability of KNN models in glioma-related tasks [14], and the specificity advantage of Ensemble models [67]. These consistencies reinforce the potential of machine learning models in accurately predicting IDH1 genotype status and highlight their role in neuro-oncological applications.

### Evaluation of radiomic features

Radiomic features have emerged as powerful noninvasive biomarkers in glioma classification, offering valuable insights into tumor heterogeneity, molecular subtypes, and prognostic outcomes. Their ability to quantify subtle variations in tumor structure and texture from imaging data has the potential to enhance diagnostic precision, guide treatment planning, and reduce the need for invasive procedures. Our study demonstrated that a combination of shape- and texture-based radiomic features played a pivotal role in distinguishing IDH1-mutant from wild-type gliomas, reinforcing the growing clinical relevance of radiomics-driven machine learning models.

Among the most influential radiomic features in our study, Low Gray Level Run Emphasis (LGLRE) and Low Gray Level Emphasis (LGLE) emerged as highly predictive of IDH1 mutation status. These findings are in line with prior research emphasizing the role of gray-level textural heterogeneity in glioma molecular profiling [64, 68]. Textural complexity has been widely associated with tumor microstructural variations, reflecting alterations in cellular density, necrosis, and microvascular proliferation, which are critical determinants of tumor grade and genetic makeup. Zhou et al. [68] reported that short-run low gray-level emphasis (SRLGLE) and run-length variance were particularly effective in IDH1 mutation prediction, achieving an AUC of 86%. The strong alignment of our findings with these studies suggests that gray-level texture analysis could serve as an imaging surrogate for IDH1 mutation detection, potentially aiding in noninvasive glioma classification.

Beyond textural complexity, morphological characteristics play a crucial role in glioma classification and prognosis. In our study, Major Axis Length (MajAL) and Surface Area were highly informative features for IDH1 classification, reflecting differences in tumor geometry that may correlate with infiltrative tumor growth and aggressiveness. These findings are consistent with those of Cao et al. [7], who demonstrated that combining shape-based radiomic features with structured imaging descriptors significantly improved IDH1 mutation prediction, achieving an AUC of 0.879. Clinically, tumors with irregular, elongated, or larger surface areas often exhibit more aggressive behavior and may be associated with poorer outcomes. The integration of shape-based radiomics into IDH1 classification models could therefore provide additional diagnostic insights, allowing clinicians to stratify patients based on noninvasive imaging biomarkers and tailor therapeutic approaches accordingly.

The clinical utility of radiomic features extends beyond molecular characterization to prognostic assessment and treatment response prediction. Prior research has demonstrated that GLSZM-derived metrics and zone% can predict tumor progression and treatment response following Gamma Knife radiosurgery (GKRS) in brain metastases [14, 21]. Our study found that GLSZM-based features played a similarly crucial role in IDH1 classification, suggesting that these metrics may not only differentiate molecular subtypes but also offer prognostic insights into tumor behavior and treatment sensitivity. In clinical practice, this could aid in identifying patients who may benefit from targeted therapies or aggressive management strategies.

Additionally, while static radiomic features provide a snapshot of tumor characteristics at a given timepoint, the integration of delta-radiomics, which captures longitudinal imaging changes, holds promise for dynamic monitoring of gliomas. Jeong et al. [24] reported that delta-radiomic features extracted from DSC-MRI could distinguish high- and low-grade gliomas with an accuracy of 96%, reinforcing the importance of tracking tumor evolution over time. Although our study did not incorporate DSC-MRI-derived features, the strong predictive value of gray-level texture and shape-based radiomics suggests that future studies incorporating longitudinal imaging biomarkers could further refine glioma classification and prognostic modeling.

### Key findings and clinical significance

Machine learning and radiomics have demonstrated strong potential as noninvasive tools in glioma classification, offering improved diagnostic accuracy and clinical utility. Studies focusing on differentiating glioblastoma from brain metastasis, glioma grading, MGMT methylation status prediction, and tumor recurrence localization have consistently employed models such as RF, CNN, and SVM, achieving sensitivity values ranging from 66 to 91.67%, specificity from 66 to 98.75%, accuracy from 67 to 98.1%, and AUC values between 0.646 and 0.992 [2, 7, 9, 16, 17, 56, 61, 62]. These findings highlight the robustness of machine learning models in noninvasive, imaging-based glioma classification, reinforcing their potential as reliable tools in clinical neuro-oncology.

Our study builds upon this foundation by demonstrating that gray-level texture and shape-based radiomic features are particularly effective in distinguishing IDH1-mutant from wild-type gliomas. Specifically, features such as Low Gray Level Run Emphasis (LGLRE) and Low Gray Level Emphasis (LGLE) emerged as highly predictive, aligning with previous reports that emphasize the role of textural heterogeneity in glioma molecular characterization [7, 21, 24, 64, 68]. Furthermore, our findings suggest that integrating radiomic features into clinical workflows could provide a noninvasive alternative to biopsy-based molecular testing, enhance patient stratification, and optimize treatment planning. Given the growing clinical reliance on imaging-based diagnostics, future efforts should focus on standardizing radiomic feature extraction, incorporating multimodal imaging techniques, and validating these models across larger patient cohorts to enhance their applicability and reliability in real-world clinical settings.

In heterogeneous tumors like gliomas, accurate classification of molecular and genetic subtypes can have a direct impact on treatment decisions. In our study, we analyzed the IDH1 genotype, verified through histopathology, because IDH1 mutation is associated with a more favorable prognosis in LGGs and serves as a critical marker for understanding tumor biology. Detecting the IDH1 mutation enables the molecular classification of gliomas and plays an essential role in predicting patient survival outcomes. Without this validation, the clinical and biological implications of imaging findings may remain limited; thus, histopathological analysis is crucial for enhancing the accuracy of noninvasive predictive models. Additionally, histopathological validation is vital for evaluating tumor progression, recurrence, pseudoprogression, and radionecrosis, as well as for assessing treatment response. Identifying IDH1 mutations provides deeper insights into treatment sensitivity and tumor behavior, facilitating the selection of appropriate therapeutic strategies. Therefore, histopathological confirmation of markers like IDH1 mutation not only reinforces predictive model reliability, bolsters diagnostic decision-making, and contributes to glioma treatment management [3, 12, 16, 61].

### Challenges in model performances

In this study, although high accuracy and sensitivity levels were achieved, the low specificity values observed across all models highlight a tendency to incorrectly classify negative cases as positive, warranting further examination. This pattern was particularly evident in models such as KNN and ensemble-based classifiers, which achieved 100% sensitivity but only 27.27% specificity, resulting in a high false-positive rate. Such imbalanced classification poses a significant challenge, particularly in clinical applications where false positives can lead to unnecessary interventions. Given this trade-off, multiple approaches were explored to mitigate specificity limitations, including class balancing techniques, threshold adjustment, and weighted loss functions [11, 18].

In this study, although the distribution of IDH1-positive and IDH1-negative cases is relatively balanced (49.1% vs. 50.9%), SMOTE was still applied to artificially increase the representation of the minority class. However, despite successfully increasing sensitivity, SMOTE did not lead to a meaningful improvement in specificity, with false-positive rates remaining high across all models. This result aligns with previous studies, such as Sakai et al. [54], who implemented SMOTE for IDH1 classification and observed only a marginal impact on specificity. These findings suggest that while oversampling methods enhance model sensitivity, they do not necessarily resolve the inherent trade-off between sensitivity and specificity in glioma molecular classification.

As an alternative, weighted loss functions were employed to explore their impact on specificity. This approach effectively increased specificity across all models; however, it also introduced significant trade-offs. Tree-based classifiers exhibited near-perfect classification performance, which was likely a result of overfitting rather than true generalizability. Conversely, models such as LR and SVM suffered a complete loss of sensitivity, failing to identify any positive cases. These findings reinforce that modifying loss functions alone is not a universally effective solution, as increasing specificity often comes at the cost of sensitivity.

To refine the balance between these metrics, threshold adjustment was applied, allowing for a more controlled classification of positive and negative cases. By varying the classification threshold, it was observed that increasing the threshold led to a significant rise in specificity while simultaneously reducing sensitivity. While lower thresholds enhanced sensitivity by capturing more positive cases, they also increased the false-positive rate. Conversely, higher thresholds minimized false positives but resulted in the complete misclassification of some positive cases. This is consistent with prior research; for instance, Lohmann et al. [38] explored threshold modifications in a FET-PET radiomics model for tumor differentiation but still reported a specificity as low as 40%, indicating that threshold adjustments alone may not fully resolve false-positive classification issues. These findings suggest that threshold optimization can serve as a flexible tool to fine-tune model performance, but it is not a standalone solution. Instead, combining it with specificity-targeted hyperparameter tuning and feature selection may be necessary to achieve a more balanced trade-off between sensitivity and specificity.

Another factor contributing to the low specificity values may be the inherent heterogeneity and radiogenetic variability in gliomas, making it challenging to distinguish between IDH1-positive and IDH1-negative cases consistently. Several prior studies have reported similar difficulties in achieving high specificity in glioma classification tasks, even when using advanced machine learning models. Kihira et al. [29] demonstrated that while their U-Net-based glioma segmentation model achieved high sensitivity, specificity remained low due to overlapping radiomic features. Ye et al. [66] explored a 3D multimodal CNN approach for glioma grading and observed that while sensitivity was consistently high, specificity varied significantly across MRI modalities (FLAIR: 41.8%, T1: 37.0%, T2: 44.0%). Liu et al. [36] further reinforced this trend, showing that SVM model achieved 85.7% sensitivity but 0% specificity, highlighting the persistent challenge of reducing false positives in glioma classification.

This trend aligns with our findings, where KNN and ensemble models demonstrated high sensitivity at the cost of low specificity, reinforcing that the trade-off between sensitivity and specificity remains a significant challenge in machine learning-based glioma classification. These observations suggest that achieving a balanced sensitivity-specificity trade-off remains a critical issue and highlights the need for continued methodological refinements. Future studies should explore multi-modal data integration, advanced feature engineering, and specificity-driven model tuning to optimize glioma classification performance in a clinically meaningful way.

### Trade-off analysis of machine learning models for IDH1 genotype classification in gliomas

Machine learning models exhibit varying trade-offs in terms of sensitivity, specificity, computational efficiency, and clinical applicability when used for IDH1 genotype classification in gliomas. The selection of the most suitable model depends on clinical priorities, such as whether the primary goal is to ensure early detection of IDH1 mutations or to minimize misclassification of IDH1-wildtype tumors.

#### Sensitivity, specificity, and clinical considerations

A critical trade-off in this study was between sensitivity—the ability to correctly detect IDH1-mutant gliomas—and specificity, which determines how well IDH1-wildtype gliomas are distinguished. In a clinical setting, this distinction has significant implications for treatment decisions, prognosis estimation, and therapeutic planning.

##### High-sensitivity models: minimizing false negatives in IDH1-mutant cases

The Ensemble (Boosted Trees) and KNN models achieved 100% sensitivity, ensuring no IDH1-mutant cases were misclassified as wildtype. Clinically, this is crucial for treatment selection, as IDH1-mutant gliomas have a more favorable prognosis and often require different therapeutic approaches than IDH1-wildtype tumors. However, these models exhibited lower specificity, leading to a higher false positive rate. This means that some IDH1-wildtype gliomas may be misclassified as mutant, potentially leading to inappropriate treatment decisions.

##### Balanced models: optimizing sensitivity and specificity for clinical accuracy

SVM and DT models provided a more balanced classification, with high sensitivity while maintaining moderate specificity. These models may be preferred in cases where both accurate detection of IDH1-positive gliomas and minimizing false positives are equally important for example, in pre-surgical planning, where an incorrect classification could affect surgical decisions. DT models are particularly advantageous in clinical environments where model interpretability is crucial, as they offer transparent decision rules that physicians can directly interpret.

##### High-specificity models: minimizing false positives in IDH1-wildtype cases

LR and Discriminant Analysis exhibited the weakest overall performance, particularly in distinguishing IDH1-mutant from wildtype gliomas. These models had higher specificity but lower sensitivity, meaning that while they reduced false positives, they risked failing to detect IDH1-positive gliomas. In a clinical setting, this could be problematic because missing an IDH1-mutant tumor might result in suboptimal treatment strategies. Thus, from a clinical perspective, models with higher sensitivity, such as Ensemble and KNN, may be preferable when the primary goal is to detect all IDH1-mutant cases, even at the cost of some false positives. Conversely, in cases where accurate differentiation between wildtype and mutant tumors is essential, balanced models such as SVM and DT are more appropriate.

#### Computational efficiency and clinical feasibility

Beyond classification accuracy, computational efficiency and real-world clinical applicability also influence the choice of machine learning models.

##### Clinically deployable models: fast, interpretable, and scalable approaches

DT and LR are computationally efficient and easily interpretable, making them suitable for real-time clinical decision support systems. However, DT models are prone to overfitting, which may limit their generalization to unseen cases.

##### High-accuracy models: computationally intensive but effective

SVM and Ensemble methods are more robust to high-dimensional radiomics data, making them better suited for complex glioma classification tasks. However, SVM requires extensive hyperparameter tuning, and Ensemble methods are computationally expensive, making them less practical for immediate clinical application without high-performance computing resources.

##### Instance-based models: scalability challenges in large-scale applications

KNN showed excellent classification performance, but its computational cost increases significantly with larger datasets. Unlike parametric models that learn a fixed decision boundary, KNN must perform real-time distance calculations for each new patient sample, making it less scalable for large-scale hospital deployments.

### Clinical utility of machine learning in glioma diagnosis

Radiomics and machine learning-based classification approaches have demonstrated significant potential in glioma diagnosis by providing non-invasive alternatives to traditional biopsy-driven molecular subtyping. While the primary advantage of radiomics lies in its ability to extract high-dimensional imaging features that capture tumor heterogeneity, the clinical applicability of these models requires further consideration. Machine learning-based models should not be viewed as standalone diagnostic tools but rather as decision-support systems that augment radiologists’ evaluations rather than replacing them [15, 34].In routine clinical practice, radiologists interpret MRI scans based on qualitative assessments of tumor shape, contrast enhancement, and signal intensity patterns. However, these evaluations can be subjective and influenced by interobserver variability [20, 41]. Machine learning models complement radiologists’ expertise by quantitatively analyzing tumor heterogeneity, identifying subtle imaging patterns that may not be visually perceptible, and reducing diagnostic inconsistencies. For example, in cases where gliomas exhibit ambiguous imaging features, ML models can flag tumors with radiomic signatures highly indicative of IDH1 mutation, prompting radiologists to consider additional molecular testing or closer follow-up.

Another key advantage of integrating ML into radiology workflows is the potential for automated risk stratification. Radiomics-based machine learning models can help prioritize high-risk cases by predicting tumor aggressiveness and progression likelihood, allowing radiologists to allocate more attention to challenging cases [28]. Additionally, ML models can serve as secondary readers by cross-checking radiologists’ initial assessments, identifying discrepancies, and suggesting alternative interpretations, particularly in borderline cases where IDH1 mutation status is uncertain.

Despite these benefits, clinical adoption of ML-driven radiomics requires careful validation to ensure reliability and interpretability. Unlike deep learning-based approaches that operate as “black-box” systems, traditional ML models provide explicit feature importance rankings, allowing radiologists to understand which imaging biomarkers contribute most to classification [1]. This level of transparency is crucial for building trust in AI-assisted diagnostics, as it enables clinicians to verify predictions and integrate ML findings with conventional diagnostic criteria.

Furthermore, machine learning models can assist in streamlining radiology workflows by automating certain repetitive tasks, such as tumor segmentation and volumetric analysis. By reducing the time required for manual measurements, ML-powered tools can enhance radiologists’ efficiency while maintaining diagnostic accuracy. In multidisciplinary tumor boards, where treatment plans are discussed, ML-driven risk assessments can provide additional objective insights, facilitating more personalized treatment decisions [37].

Ultimately, the successful implementation of ML in glioma diagnosis depends on seamless integration into existing clinical workflows rather than replacing human expertise. These models should function as assistive tools that enhance decision-making, improve diagnostic consistency, and optimize patient management rather than as standalone diagnostic entities. Future studies should focus on prospective validation of ML-assisted workflows in real-world radiology settings to assess their clinical impact and usability.

However, while machine learning offers substantial benefits in tumor classification, its performance is highly dependent on the accuracy of tumor segmentation. Given that tumor delineation is a crucial step in radiomics-based glioma classification, the choice between manual and deep learning-based segmentation methods remains an essential consideration in clinical implementation.

### Manual vs. deep learning-based segmentation in glioma classification

In glioma research, both manual segmentation and deep learning-based segmentation techniques have been widely employed for tumor delineation. Manual segmentation, as utilized in this study, involves expert-driven annotation of tumor regions, ensuring precise and reproducible tumor boundaries. In contrast, deep learning-based segmentation, primarily using convolutional neural networks (CNNs), offers an automated alternative that eliminates the need for manual delineation [22, 48].

Manual segmentation remains the gold standard for radiomic feature extraction due to its high accuracy and consistency in controlled settings. Expert-driven segmentation ensures that tumor boundaries are carefully delineated based on radiological knowledge, reducing errors caused by misclassification of peritumoral edema or necrotic regions. In glioma classification studies, manual segmentation is particularly valuable in small datasets, where variations in tumor delineation could significantly impact model performance [5, 48].

However, manual segmentation is time-consuming, labor-intensive, and subject to interobserver variability [41]. Despite rigorous quality control, minor differences in annotation between experts can introduce variability in radiomic feature extraction. This is a key limitation when working with large-scale datasets or developing AI-driven automated classification models for real-time clinical applications.

Deep learning-based segmentation, particularly using CNNs, has gained popularity due to its ability to automatically extract tumor regions with high precision. Studies have demonstrated that CNN-based models can segment gliomas with high Dice similarity coefficients, improving workflow efficiency in clinical settings [5, 22]. Deep learning models eliminate the need for manual intervention, making them suitable for large-scale studies and reducing segmentation variability across different institutions.

Despite these advantages, CNN-based segmentation presents several challenges in glioma classification. CNN-based segmentation models require large, well-annotated training datasets to achieve high accuracy. However, gliomas are relatively rare tumors, and manually labeled datasets with precise tumor annotations are limited. Unlike other cancers, gliomas exhibit significant heterogeneity, making it difficult to develop generalized deep learning models without extensive training data [41].

CNN-based segmentation models operate with complex internal mechanisms that do not explicitly reveal how specific imaging features contribute to segmentation, making it challenging for radiologists to directly interpret the decision-making process. In contrast, radiomics-based machine learning models allow explicit feature extraction, providing transparency regarding which imaging biomarkers contribute most to classification [1].

Implementing deep learning-based segmentation requires high computational power and complex hyperparameter tuning. In contrast, manual segmentation ensures precise tumor delineation without the need for GPU-intensive processing, making it more practical in standard clinical settings [5].

CNN-based segmentation models trained on one dataset may not generalize well to other datasets due to differences in imaging protocols, scanner models, and institutional variations. Radiomics-based models, which rely on standardized feature extraction, offer greater flexibility in adapting to different clinical settings [35].

Given the relatively small dataset used in this study, manual segmentation was preferred to ensure accurate, high-quality tumor delineation. Unlike CNN-based segmentation, which requires extensive training data and hyperparameter optimization, manual segmentation provided a controlled and reliable method for radiomic feature extraction. By maintaining precise tumor boundaries, this approach minimized the risk of segmentation-induced feature variations, thereby improving model stability.

Additionally, since this study focuses on radiomics-based IDH1 classification, the interpretability of radiomic features is a critical factor. Deep learning models do not inherently provide explicit feature importance rankings, whereas traditional machine learning algorithms allow for transparent identification of the most relevant radiomic features [1].

While this study relied on manual segmentation, future research could explore hybrid approaches that combine CNN-based segmentation with radiomics-based ML models. Studies have shown that integrating deep learning segmentation with handcrafted radiomic features can improve classification accuracy while maintaining interpretability [6]. Such an approach would allow for the automation of tumor segmentation while preserving the transparency and robustness of radiomics-based classification.

### Strengths of the study

The machine learning-based radiomic analyses employed in our study eliminate the physical and psychological burden associated with biopsy procedures due to their non-invasive nature. These methods make the diagnostic process less stressful and reduce hospital stays, thereby contributing to patient safety and comfort. Radiomic analyses evaluate the entire tumor using cross-sectional MRI imaging, capturing its heterogeneity. This approach enables a more reliable genotypic classification compared to single-region biopsy samples, facilitating a more comprehensive diagnosis. Furthermore, the rapid processing of imaging data in radiomic analyses accelerates diagnostic workflows. While waiting for biopsy results can take days or weeks, radiomic analyses can deliver results within minutes or hours, offering both speed and efficiency.

Biopsy procedures require surgical equipment, specialized expertise, and appropriate hospital settings, whereas radiomic analyses rely on imaging data obtained through standard imaging protocols. Radiomic methods are especially beneficial in areas where advanced surgical facilities are not readily available. Machine learning-based radiomic analyses thus provide advantages in accessibility. Additionally, these analyses enhance diagnostic and treatment planning by integrating molecular and radiological data to classify glioma subtypes more effectively. This integration optimizes the prognostic and predictive value of IDH1 mutations, enabling risk stratification and more personalized treatment planning.

### Limitations of the study

Apart from the challenges associated with specificity, our study has other limitations. First, our analyses were conducted on a relatively limited patient population. The size of our dataset may constrain the generalizability of the models and underscores the need for validation in larger, multi-center populations.

Second, radiomic analyses can be influenced by the technical parameters and protocols of the imaging devices used. In this study, only specific MRI protocol was employed; thus, our results may not be directly generalizable to other devices or protocols. This highlights the necessity for harmonization of radiomic features across different imaging systems.

Third, the segmentation process was performed using manual method. This introduces the potential for inter-observer variability, which could affect the accuracy of the analyses. Employing fully automated segmentation methods in future studies could mitigate this limitation and provide more reproducible results.

Finally, the performance of machine learning models depends on the optimization of hyperparameter settings and the accuracy of selected features. While these parameters were carefully tuned in this study, exploring different models and modeling approaches could be beneficial for result comparison. Moreover, the biological relevance of the radiomic features used to identify the IDH1 genotype should be further investigated in subsequent research.

Despite these limitations, our study demonstrates the potential of radiomic and machine learning approaches as noninvasive tools for molecular classification in gliomas, providing a valuable foundation for future research. Radiomics and machine learning models offer non-invasive alternatives to traditional invasive diagnostic approaches, providing significant contributions to clinical practice.

Although invasive biopsy remains the gold standard for obtaining molecular-level information in glioma diagnosis, it has several limitations and challenges. Biopsy procedures often require surgical intervention and carry significant risks of complications. Brain biopsies, in particular, are associated with potential adverse effects such as intracranial hemorrhage, infection, and neurological deficits. Moreover, biopsy samples represent only a specific portion of the tumor rather than its entirety, limiting their ability to reflect tumor heterogeneity. This limitation is particularly critical given the biological diversity of gliomas, which may lead to misclassification or incomplete clinical information.

## Conclusion

This study demonstrates that machine learning-based radiomic models provide a reliable and noninvasive approach to IDH1 genotype classification in gliomas. Among the models tested, KNN exhibited the highest classification performance with an AUC-ROC of 0.90 and 100% sensitivity, reinforcing its potential as an effective tool for molecular subtyping. Additionally, shape- and texture-based radiomic features, particularly LGLRE and LGLE, emerged as strong predictors, highlighting the role of tumor heterogeneity in glioma characterization.

The integration of machine learning into clinical workflows could significantly enhance glioma diagnosis and treatment planning by offering an automated, rapid, and objective alternative to biopsy-based molecular testing. These models could aid in early decision-making, particularly in cases where biopsy is not feasible due to tumor location or patient condition. Furthermore, machine learning has the potential to reduce diagnostic turnaround time, minimize interobserver variability, and complement traditional histopathological evaluation, improving overall patient management.

Future research should focus on validating these models in multi-center datasets, refining specificity through feature optimization, and integrating multimodal imaging approaches. Advancements in machine learning-based radiomics are expected to make it a fundamental tool in precision neuro-oncology and an integral part of glioma diagnosis and management.

## Electronic supplementary material

Below is the link to the electronic supplementary material.

Supplementary Material 1

## Data Availability

No datasets were generated or analysed during the current study.
