# Supplementary material for: From pixels to prognosis: Leveraging radiomics and machine learning to predict IDH1 genotype in gliomas
Source: Neurosurg Rev. 2025 Apr 29;48(1):396. doi: 10.1007/s10143-025-03515-z (PMC12040993; doi:10.1007/s10143-025-03515-z)
Supplement: Supplementary file 1 — Supplementary Material 1 [file 10143_2025_3515_MOESM1_ESM.docx]

**Table S1.** Description and calculation methods of 112 radiomic features.

| **No** | **Feature** | **Description** | **Category** |
| --- | --- | --- | --- |
| **1** | Mean | Average voxel intensity within the ROI. | First-Order (FOS) |
| **2** | Minimum | Minimum voxel intensity value within the ROI. | First-Order (FOS) |
| **3** | Maximum | Maximum voxel intensity value within the ROI. | First-Order (FOS) |
| **4** | VoxelNum | Number of voxels within the ROI. | Shape-Based (Shape) |
| **5** | VolumeNum | Volume of the tumor region in voxels. | Shape-Based (Shape) |
| **6** | Elongation | Describes the elongation of the tumor's shape. | Shape-Based (Shape) |
| **7** | Flatness | Quantifies how flat the object is. | Shape-Based (Shape) |
| **8** | LeastAxisLength | Length of the shortest axis of the bounding box. | Shape-Based (Shape) |
| **9** | MajorAxisLength | Length of the longest axis of the bounding box. | Shape-Based (Shape) |
| **10** | Maximum2DDiameterColumn | Maximum diameter of the tumor in column. | Shape-Based (Shape) |
| **11** | Maximum2DDiameterRow | Maximum diameter of the tumor in row. | Shape-Based (Shape) |
| **12** | Maximum2DDiameterSlice | Maximum diameter of the tumor in a slice. | Shape-Based (Shape) |
| **13** | Maximum3DDiameter | Maximum 3D diameter of the tumor. | Shape-Based (Shape) |
| **14** | MeshVolume | Volume of the tumor mesh. | Shape-Based (Shape) |
| **15** | MinorAxisLength | Length of the minor axis of the bounding box. | Shape-Based (Shape) |
| **16** | Sphericity | Sphericity of the tumor shape. | Shape-Based (Shape) |
| **17** | SurfaceArea | Surface area of the tumor. | Shape-Based (Shape) |
| **18** | SurfaceVolumeRatio | Ratio of surface area to volume. | Shape-Based (Shape) |
| **19** | VoxelVolume | Volume of a single voxel in mm³. | First-Order (FOS) |
| **20** | X10Percentile | 10th percentile of voxel intensities. | First-Order (FOS) |
| **21** | X90Percentile | 90th percentile of voxel intensities. | First-Order (FOS) |
| **22** | Energy | Sum of squared voxel intensities. | First-Order (FOS) |
| **23** | Entropy | Measure of randomness in the intensity distribution. | First-Order (FOS) |
| **24** | InterquartileRange | Range of the middle 50% of voxel intensities. | First-Order (FOS) |
| **25** | Kurtosis | Measure of peakedness in the intensity distribution. | First-Order (FOS) |
| **26** | Skewness | Measure of asymmetry in the intensity distribution. | First-Order (FOS) |
| **27** | Autocorrelation | Measure of voxel intensity correlation. | GLCM |
| **28** | ClusterProminence | Quantifies sharpness in intensity clustering. | GLCM |
| **29** | ClusterShade | Measures asymmetry in voxel intensity clustering. | GLCM |
| **30** | ClusterTendency | Tendency of voxel intensities to form clusters. | GLCM |
| **31** | Contrast | Measure of local contrast within the ROI. | GLCM |
| **32** | Correlation | Linear dependence between intensities. | GLCM |
| **33** | DifferenceAverage | Average of differences between neighboring voxel intensities. | GLCM |
| **34** | DifferenceEntropy | Measure of randomness in the difference between voxel intensities. | GLCM |
| **35** | DifferenceVariance | Variance of the differences between voxel intensities. | GLCM |
| **36** | Id (Inverse Difference) | Measure of intensity homogeneity. | GLCM |
| **37** | Idm (Inverse Difference Moment) | Emphasizes homogeneity of neighboring voxels. | GLCM |
| **38** | Idmn (Inverse Difference Normalized) | Normalized measure of homogeneity in voxel intensities. | GLCM |
| **39** | Idn (Inverse Difference Normalized 2) | Alternative normalized homogeneity measure. | GLCM |
| **40** | Imc1 (Informational Measure of Correlation 1) | Captures correlation between intensity distributions. | GLCM |
| **41** | Imc2 (Informational Measure of Correlation 2) | Second-order correlation measure. | GLCM |
| **42** | JointAverage | Average intensity of paired voxel values. | GLCM |
| **43** | JointEnergy | Sum of squared probabilities of intensity pairs. | GLCM |
| **44** | JointEntropy | Entropy of paired voxel intensities. | GLCM |
| **45** | MCC (Maximal Correlation Coefficient) | Maximum correlation between voxel intensity distributions. | GLCM |
| **46** | MaximumProbability | Maximum probability of a voxel intensity pair. | GLCM |
| **47** | SumAverage | Average of sums of voxel intensity pairs. | GLCM |
| **48** | SumEntropy | Entropy of summed voxel intensity pairs. | GLCM |
| **49** | SumSquares | Second moment of voxel intensities. | GLCM |
| **50** | DependenceEntropy | Randomness in gray-level dependence distribution. | GLDM |
| **51** | DependenceNonUniformity | Non-uniformity of gray-level dependence distribution. | GLDM |
| **52** | DependenceNonUniformityNormalized | Normalized dependence non-uniformity. | GLDM |
| **53** | DependenceVariance | Variance of dependence in gray levels. | GLDM |
| **54** | GrayLevelNonUniformity | Non-uniformity in gray-level distribution. | GLDM |
| **55** | GrayLevelVariance | Variance of gray levels in the ROI. | GLDM |
| **56** | HighGrayLevelEmphasis | Emphasis on high gray-level intensities. | GLDM |
| **57** | LargeDependenceEmphasis | Emphasis on large dependencies in gray-level intensities. | GLDM |
| **58** | LargeDependenceHighGrayLevelEmphasis | Emphasis on large regions with high gray levels. | GLDM |
| **59** | LargeDependenceLowGrayLevelEmphasis | Emphasis on large regions with low gray levels. | GLDM |
| **60** | LowGrayLevelEmphasis | Emphasis on low gray-level intensities. | GLDM |
| **61** | SmallDependenceEmphasis | Emphasis on small dependencies in gray-level intensities. | GLDM |
| **62** | SmallDependenceHighGrayLevelEmphasis | Emphasis on small regions with high gray levels. | GLDM |
| **63** | SmallDependenceLowGrayLevelEmphasis | Emphasis on small regions with low gray levels. | GLDM |
| **64** | GrayLevelNonUniformity_A | Non-uniformity in gray-level distribution for region A. | GLDM |
| **65** | GrayLevelNonUniformityNormalized | Normalized non-uniformity of gray levels. | GLDM |
| **66** | GrayLevelVariance_A | Variance of gray levels in region A. | GLDM |
| **67** | HighGrayLevelRunEmphasis | Emphasis on high gray-level intensities in runs. | GLRLM |
| **68** | LongRunEmphasis | Emphasis on long continuous runs of voxel intensities. | GLRLM |
| **69** | LongRunHighGrayLevelEmphasis | Emphasis on high gray levels in long intensity runs. | GLRLM |
| **70** | LongRunLowGrayLevelEmphasis | Emphasis on low gray levels in long intensity runs. | GLRLM |
| **71** | LowGrayLevelRunEmphasis | Emphasis on low gray-level intensities in runs. | GLRLM |
| **72** | RunEntropy | Entropy of voxel intensity runs. | GLRLM |
| **73** | RunLengthNonUniformity | Non-uniformity of run lengths. | GLRLM |
| **74** | RunLengthNonUniformityNormalized | Normalized non-uniformity of run lengths. | GLRLM |
| **75** | RunPercentage | Percentage of total voxel runs in the ROI. | GLRLM |
| **76** | RunVariance | Variance in the lengths of voxel intensity runs. | GLRLM |
| **77** | ShortRunEmphasis | Emphasis on short continuous runs of voxel intensities. | GLRLM |
| **78** | ShortRunHighGrayLevelEmphasis | Emphasis on high gray levels in short intensity runs. | GLRLM |
| **79** | ShortRunLowGrayLevelEmphasis | Emphasis on low gray levels in short intensity runs. | GLRLM |
| **80** | GrayLevelNonUniformity_B | Non-uniformity in gray-level distribution for region B. | GLRLM |
| **81** | GrayLevelNonUniformityNormalized_A | Normalized non-uniformity of gray levels in region A. | GLRLM |
| **82** | GrayLevelVariance_B | Variance of gray levels in region B. | GLRLM |
| **83** | HighGrayLevelZoneEmphasis | Emphasis on high gray-level intensities in zones. | GLSZM |
| **84** | LargeAreaEmphasis | Emphasis on large areas of continuous intensity zones. | GLSZM |
| **85** | LargeAreaHighGrayLevelEmphasis | Emphasis on high gray levels in large intensity areas. | GLSZM |
| **86** | LargeAreaLowGrayLevelEmphasis | Emphasis on low gray levels in large intensity areas. | GLSZM |
| **87** | LowGrayLevelZoneEmphasis | Emphasis on low gray-level intensities in zones. | GLSZM |
| **88** | SizeZoneNonUniformity | Non-uniformity in the size of intensity zones. | GLSZM |
| **89** | SizeZoneNonUniformityNormalized | Normalized non-uniformity in the size of intensity zones. | GLSZM |
| **90** | SmallAreaEmphasis | Emphasis on small areas of continuous intensity zones. | GLSZM |
| **91** | SmallAreaHighGrayLevelEmphasis | Emphasis on high gray levels in small intensity areas. | GLSZM |
| **92** | SmallAreaLowGrayLevelEmphasis | Emphasis on low gray levels in small intensity areas. | GLSZM |
| **93** | ZoneEntropy | Entropy of intensity zones. | GLSZM |
| **94** | ZonePercentage | Percentage of total intensity zones in the ROI. | GLSZM |
| **95** | ZoneVariance | Variance in the sizes of intensity zones. | GLSZM |
| **96** | Busyness | Measure of the complexity of voxel intensity transitions. | NGTDM |
| **97** | Coarseness | Measure of intensity variations across the ROI. | NGTDM |
| **98** | Complexity | Quantifies the level of detail in intensity distribution. | NGTDM |
| **99** | Contrast_A | Local intensity contrast measure for region A. | NGTDM |
| **100** | Strength | Measure of the robustness of intensity transitions in the ROI. | NGTDM |
| **101** | GrayLevelNonUniformity_B | Non-uniformity in gray-level distribution. | GLSZM |
| **102** | HighGrayLevelRunEmphasis | Emphasis on high-intensity values in runs. | GLRLM |
| **103** | ShortRunHighGrayLevelEmphasis | Short runs of high-intensity values. | GLRLM |
| **104** | LongRunHighGrayLevelEmphasis | Long runs of high-intensity values. | GLRLM |
| **105** | LowGrayLevelZoneEmphasis | Focus on lower gray-level zones. | GLSZM |
| **106** | LargeDependenceLowGrayLevelEmphasis | Large dependence on low values. | GLDM |
| **107** | SmallAreaEmphasis | Smaller voxel relationships in ROIs. | GLSZM |
| **108** | SmallDependenceHighGrayLevelEmphasis | High-dependency zones with high-level data. | GLDM |
| **109** | DependenceNonUniformityNormalized | Dependence zone variability. | GLDM |
| **110** | SmallDependenceLowGrayLevelEmphasis | Compact zones with low-intensity runs. | GLDM |
| **111** | ZoneEntropy | Total zone-level entropy distribution. | GLSZM |
| **112** | Strength | Comprehensive region strength and homogeneity. | NGTDM |
